# 2-O-*β*-d-glucopyranosyl-_l_-ascorbic acid, a novel vitamin C derivative from *Lycium barbarum*, prevents oxidative stress

**DOI:** 10.1016/j.redox.2019.101173

**Published:** 2019-03-18

**Authors:** Shen-Fei Wang, Xin Liu, Mo-Yu Ding, Shuangcheng Ma, Jing Zhao, Ying Wang, Shaoping Li

**Affiliations:** a Institute of Chinese Medical Sciences and State Key Laboratory of Quality Research in Chinese Medicine, University of Macau, Avenida da Universidade, Taipa, Macao SAR, China; b National Institutes for Food and Drug Control, Beijing, 100050, China

**Keywords:** Ascorbic acid, 2-O-*β*-d-glucopyranosyl-_l_-ascorbic acid (AA-2*β*G), Oxidative stress, *Lycium barbarum*, Nuclear factor E2-related factor 2 (Nrf2)

## Abstract

Reducing agents are crucial for the management of maladaptive inflammation-induced macrophage death and hematopoietic toxicity of chemotherapy. 2-*O*-*β*-d-glucopyranosyl-_l_-ascorbic acid (AA-2*β*G), a unique AA (or vitamin C) derivative identified in *Lycium barbarum*, exhibited enhanced free radical scavenging activity compared with AA and its synthetic derivative AA-2*α*G. AA-2*β*G protected hydrogen peroxide-induced cell death in murine macrophage RAW264.7 cells. Treatment with AA-2*β*G eliminated oxidative stress and the ratio of cellular glutathione to glutathione disulfide more effectively than AA and AA-2*α*G. AA-2*β*G also significantly reduced the fluorescent intensity of DCFH-DA triggered by chemotherapeutic agent camptotehcin-11 but not fluorouracil. AA, AA-2*α*G, and AA-2*β*G significantly decreased Keap-1expression, and increased the expression levels of nuclear factor E2-related factor 2 (Nrf2) and heme oxygenase-1. All compounds triggered the nuclear translocation of Nrf2, while the ability of AA-2*β*G to enhance the Nrf2-DNA binding affinity was approximately two fold as those of AA and AA-2*α*G. Sodium ascorbate cotransporters (SVCT) inhibitors, sulfinpyrazone, phloretin, and 3-*O*-methyglucose, potently abrogated the free radical scavenging activities of AA, AA-2*α*G, and AA-2*β*G. The cellular uptake efficacy of AA-2*α*G and AA-2*β*G was less than 10% of AA, while the inhibition of SVCT with sulfinpyrazone considerably diminished the uptake efficacy of these compounds. AA-2*α*G and AA-2*β*G are more stable in the Fenton reagents than AA. In summary, AA-2*β*G from *L. barbarum* with excellent free radical scavenging activity is a promising natural AA derivative for further pharmacological evaluation.

## 1 Introduction

**Fig. 1:**
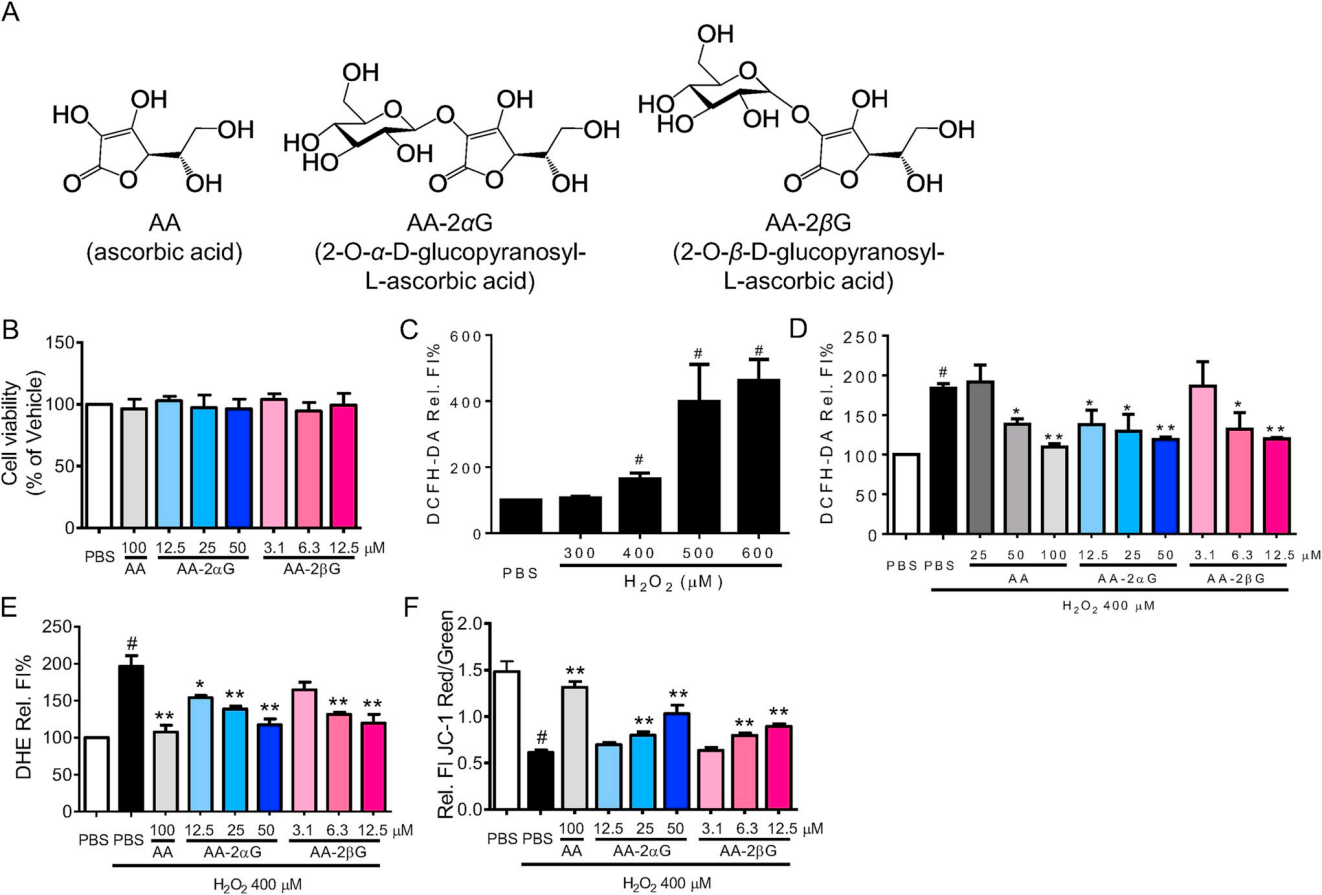
Treatment with AA, AA-2*α*G, and AA-2*β*G prevented H_2_O_2_-induced cell death in RAW264.7 cell. (A) Chemical structures of AA, AA-2*α*G, and AA-2βG. (B) RAW264.7 cell viability was measured by MTT assay after treatment with different AA, AA-2*α*G, and AA-2*β*G concentrations for 4 h. (C) RAW264.7 cells were treated with different H_2_O_2_ dosages of for 30 min. The H_2_O_2_-stimulated ROS generation was measured by DCFH-DA staining. RAW264.7 cells were pretreated with different f AA, AA-2*α*G, and AA-2*β*G concentrations for 1 h, followed by H_2_O_2_ treatment for another 30 min. Generation of ROS was analyzed by (D) DCFH-DA and (E) DHE staining, and (F) the effect on mitochondrial transmembrane potential was measured using JC-1 staining. The results are presented as mean ± SD from three separate experiments (*, *p* < 0.05 and **, *p* < 0.01 compared with H_2_O_2_ treatment; #, *p* < 0.05 compared with PBS treatment).

Imbalance between the oxidant and antioxidant system results in excess amounts of free radicals and hence oxidative stress (ROS), which is considered as a two-edged weapon. The accumulation of free radicals is associated with maladaptive inflammation-induced macrophage death that leads to the subsequent necrotic death of plaque cells [1]. Chronic elevated ROS is also responsible for hematopoietic toxicity of chemotherapeutic agents [2] and the development of many types of diseases, including various types of cancer [3] and neurodegenerative diseases [4]. Meanwhile, exclusive generation of intracellular free radicals, such as hydrogen peroxide (H_2_O_2_) and superoxide (O_2_^−^), through the oxidation of antioxidants induces apoptotic cell death in KRAS and BRAF mutant colorectal cancer [5,6]. Therefore, the pursuit of potent antioxidant reagents for pharmacological use continues.

l-Ascorbic acid (AA, or vitamin C, Fig. 1A) is one of the most widely used antioxidant in the food and cosmetic industry [5,6]. However, AA is easily oxidized to dehydrated AA (DHA) in aqueous solution [5,7]. The pharmacological but not physiology dose of AA increases the risk of kidney stone, and renal and metabolic toxicity [8]. Therefore, stable AA derivative with fast and long-lasting free radical scavenging activity and reduced therapeutic dose is urgently needed.

Medicinal plants are a rich source of antioxidants. Solanaceae family plants *Lycium barbarum* and *L. chinense* have been used as traditional Chinese Medicine for over 2,000 years [9]. The ripe fruits of *L. barbarum* and *L. chinense* or wolfberry are used to prevent or treat several types of diseases. The properties of these fruits include benefits on complexion and maintaining of beauty according to the “Compendium of Materia Medica” [10], as well as maintenance of vision [11], kidney, and liver functions [9]. Wolfberry is used as a tonic herb and functional food with antioxidant [12], anti-inflammation [13], and antiaging activities [14]. Phenolics [15], *L. barbarum* polysaccharides, β-carotenoids [16,17], or flavonoids [18] isolated from wolfberry exhibit potent radical scavenging activities. A novel AA derivative, 2-*O*-*β*-d-glucopyranosyl-L-AA (AA-2*β*G, Fig. 1A), was first purified from *L. barbarum* by Toyoda-Ono et al. in 2004 [19]. AA-2*β*G exhibited comparable antioxidant activities to AA and its synthetic derivative 2-*O*-*α*-d-glucopyranosyl-L-AA (AA-2*α*G, Fig. 1A) in cell-free 1,1-diphenyl-picrylhydrazyl radical scavenging assay [20,21]. AA-2*β*G downregulates the serum aspartate transaminase and alanine aminotransferase levels in carbon tetrachloride-induced murine hepatic damage model [22]. A comparative analysis of the radical scavenging activity and the mechanisms of action of AA, AA-2*α*G, and AA-2*β*G have not been conducted yet.

In the present study, we mainly evaluated the ROS scavenging potentials of AA, AA-2*α*G, and AA-2*β*G in M1/M2-like murine macrophage RAW264.7 cells. We also determined the effect of the d-glucosyl moiety and its configuration on cellular uptake and molecular mechanism. Our results provided the first pharmacological evidence that AA-2*β*G, the novel and stable AA derivative from *L. barbarum*, with enhanced free radical scavenging activity.

## 2 Materials and methods

### 2.1 Materials and reagents

H_2_O_2_ was purchased from Aladdin (Shanghai, China). Fluorescent probe 2′,7′-dichlorofluorescin diacetate (DCFH-DA), dihydroethidium (DHE), 5,5′,6,6′-tetrachloro-1,1′,3,3′-tetraethylbenzimidazolylcarbocyanine iodide (JC-1), AA, AA-2*α*G, camptothecin-11 (CPT-11), 5-fluorouracil (5-FU), and 3-O-methylglucose were obtained from Sigma-Aldrich (St. Louis, MO, USA). AA-2*β*G was separated and purified as previously reported [21]. Sodium-ascorbate co-transporters (SVCT) and glucose transporter inhibitors sulfinpyrazone (SU), phloretin (PH), and 3-*O*-methy glucose (3-OMG) were purchased from Cayman Chemical (Ann Arbor, MI, USA). Phosphate-buffered saline (PBS), Dulbecco's modified Eagle medium (DMEM), fetal bovine serum (FBS), and 100 × penicillin and streptomycin were purchased from Gibco-Invitrogen (Paisley, Scotland, UK). AA-2*α*G and AA-2*β*G were dissolved in PBS prior to use. Superoxide dismutase (SOD), catalase (CAT), glutathione (GSH), and glutathione disulfide (GSSG) detection kits were purchased from Beyotime Biotechnology (Shanghai, China).

### 2.2 Cell culture and treatment condition

Murine macrophage RAW 264.7 cell line was acquired from the American Type Culture Collection (ATCC, Rockville, MD, USA), and cultured in DMEM medium supplemented with 10% fetal bovine serum, 100 U/mL penicillin and streptomycin with humidified atmosphere of 5% CO_2_ at 37 °C.

For detection of SOD, CAT, GSH, GSSG, DHL, TNF-α, and western blot analysis, RAW264.7 cells were pre-incubated with AA, AA-2*α*G, or AA-2*β*G for 4 h, and then stimulated with 400 μM H_2_O_2_ for 6 h.

For co-treatment with SVCT inhibitors, RAW264.7 cells were pre-incubated with 5 μM SU, 10 μM PH, or 10 μM 3-OMG for 20 min and pre-treated with AA, AA-2*α*G, or AA-2*β*G for 4 h. ROS was then stimulated with 400 μM H_2_O_2_ for 30 min.

### 2.3 Cell viability assay

RAW 264.7 cells were seeded in 96 well plates for 18 h and then treated with AA, AA-2*α*G, AA-2*β*G, or PBS as vehicle for 4 h. MTT was then added at 5 mg/mL for an addition 4 h at 37 °C. At the end of the incubation, the formazan crystals were dissolved with DMSO. The absorbance was determined at 570 nm using MultiLabel Counter Victor (PerkinElmer, Waltham, MA, USA). Cell viability was calculated by the ratio of absorbance between treated and vehicle groups.

### 2.4 Detection of ROS and mitochondrial transmembrane potential (Δ*Ψm*)

For ROS detection, RAW 264.7 cells were pre-treated with AA, AA-2*α*G, or AA-2*β*G for 1 h, incubated with 10 μM DCFH-DA or DHE for 30 min in serum free DMEM, and then stimulated with H_2_O_2_ for 30 min. For detection of Δ*Ψm*, RAW264.7 cells were pre-treated with AA, AA-2*α*G, or AA-2*β*G for 1 h, incubated with 10 μM JC-1 at 37 °C for 30 min, and then stimulated with H_2_O_2_ for 30 min. Changes in the fluorescent intensity of DCFH-DA, DHE, and JC-1 was determined using BD Accuri™ C6 flow cytometer (BD Biosciences, San Jose, CA, USA). At least 10,000 cells were recorded for each sample. The results were analyzed by using FlowJo software (TreeStar, San Carlos, CA, USA).

### 2.5 Lactate dehydrogenase (LDH) release assay

LDH released into the culture medium was determined using LDH Diagnostic kit (Beyotime Biotechnology, Shanghai, China). The absorbance was measured at 490 nm following the manufacture's recommendation (PerkinElmer, Waltham, MA, USA).

### 2.6 Detection of extracellular tumor necrosis factor (TNF)-α level

The level of secreted TNF-α in culture supernatant was measured with Multi-Analyte Flow Assay Kit (Biolegend, San Diego, CA, USA) using BD Accuri™ C6 flow cytometer according to the manufacturer's instructions.

### 2.7 Measurement of intracellular antioxidant enzyme activity

SOD, CAT, GSH, and GSSG activities in RAW264.7 cells were measured using commercially available assay kits from Beyotime according to the manufacturer's instructions.

### 2.8 Western blot analysis

At the end of the treatment, cells were lysed with ice-cold RIPA lysis buffer (Beyotime Biotechnology) supplemented with 1% PMSF and 1% protease inhibitor cocktail (Thermo Fisher Scientific, Rockford, IL, USA) on ice for 20 min. Cell lysate were cleared by centrifugation at 12,000 rpm for 15 min at 4 °C. Protein concentration was determined using Pierce^®^ BCA Protein Assay Kit (Thermo Fisher Scientific). The samples were denatured by boiling in SDS loading buffer. Twenty μg of total protein was separated by 10% sodium dodecyl sulfate-polyacrylamide gel electrophoresis followed by electro blotting onto a polyvinylidene difluoride (PVDF) membrane (Bio-Rad, Hercules, CA, USA). After blocking in 5% nonfat milk dissolved in Tris-buffered saline-Tween 20 (TBS-T) buffer (20 mM Tris-HCl, pH 7.6, 150 mM NaCl, 0.1% Tween-20) for 1 h at room temperature, the membrane was probed with primary antibodies against Kelch-like ECH-associated protein-1 (Keap-1), nuclear factor E2-related factor 2 (Nrf2), heme oxygenase (HO)-1, and β-actin at 1:1,000 dilution in TBS-T overnight at 4 °C and specific secondary antibodies for 2 h at room temperature. Specific protein bands were visualized using ECL advanced Western blot detection kit (GE Healthcare, Uppsala, Sweden).

### 2.9 Immunofluorescent staining

Immunofluorescent analysis was performed as previously reported [23]. In brief, RAW264.7 cells were fixed with 4% paraformaldehyde for 30 min, and blocked with 3% BSA in PBS for 1 h at room temperature. Cells were then incubated with primary antibody at optimal dilution in 3% BSA at 4 °C overnight followed by fluorescent conjugated secondary antibody at 1:500 dilution. Cells were then sealed in anti-fade reagent and examined under Leica TCS SP8 confocal microscope.

### 2.10 Nrf2 DNA binding activity assay

RAW264.7 cells were pre-treated with AA, AA-2*α*G, or AA-2*β*G for 4 h, and then treated with 400 μM H_2_O_2_ for 6 h. Nuclear extract of RAW264.7 cells were prepared following the method reported previously [24]. Nrf2 promoter binding activity was measured using the Trans^AM^ Nrf2 DNA-binding ELISA assay from Active Motif (Carlsbad, CA, USA) following the manufacture's recommendation.

### 2.11 ESI-QTOF-MS analysis

Cells were washed with PBS and harvested with methanol-water (v:v, 80:20). Equal number of cells were lysed by sonication and cleared by centrifugation at 15,000 rpm for 20 min at 4 °C. The intracellular content of AA, AA-2*α*G, or AA-2*β*G were quantitatively analyzed using ESI-Q-TOF-MS/MS on a Bruker Impact HD Q-TOF MS system operating in ESI positive ion mode. The mass detection range was set as *m/z* 100–1500 in the full scan mode. Samples were injected into the system and quantify with external standard dissolved in methanol-water (v:v, 80:20).

### 2.12 *In vitro* redox reaction

*In vitro* redox reaction was conducted with Fenton reagents [25]. In brief, 100 μL of Fenton reagents was added to equal volume of AA, AA-2*α*G, or AA-2*β*G in PBS. The change of absorbance was measured at 300 nm using FlexStation 5 multi-mode microplate reader (Molecular Devices, San Jose, CA, USA) every 3 min for a total of 30 min.

### 2.13 Statistical analysis

Data were presented as mean ± SD from at least three independent experiments. Statistical significance between different experimental groups was determined by one-way analysis of variance (ANOVA) followed by Dunnett's post hoc test, and *p* values less than 0.05 was considered statistically significant.

## 3 Results

### 3.1 AA and its derivatives exhibited radical scavenging activity

We used H_2_O_2_-induced oxidative stress in murine M1/M2 macrophage RAW264.7 cells to determine the antioxidant activities of AA, AA-2*α*G, and AA-2*β*G. None of these compounds alone affect cell viability as detected using MTT assay (Fig. 1B). Then, we then stimulated RAW264.7 cells with different concentrations of H_2_O_2_, and found that H_2_O_2_ at 400 μM began to trigger ROS considerably after 30 min (Fig. 1C). AA, AA-2*α*G, and AA-2*β*G significantly reduced the DCFH-DA oxidation in H_2_O_2_-treated RAW264.7 cells in a dose-dependent manner (Fig. 1D). AA-2*α*G at 50 μM and AA-2*β*G at 12.5 μM suppressed the H_2_O_2_-induced fluorescence of oxidized DHE to the same extent as 100 μM AA (Fig. 1E). Treatment with H_2_O_2_ decreased the ratio of JC-1 red to JC-1 green. Meanwhile, AA, AA-2*α*G, and AA-2*β*G potently enhanced the ratio of JC-1 read to JC-1 green, thereby indicating that H_2_O_2_-reduced Δ*Ψm* was restored by these compounds (Fig. 1F).

### 3.2 Treatments with AA, AA-2*α*G, and AA-2*β*G restored intracellular antioxidant enzyme activity and abrogated H_2_O_2_-induced cell death

**Fig. 2:**
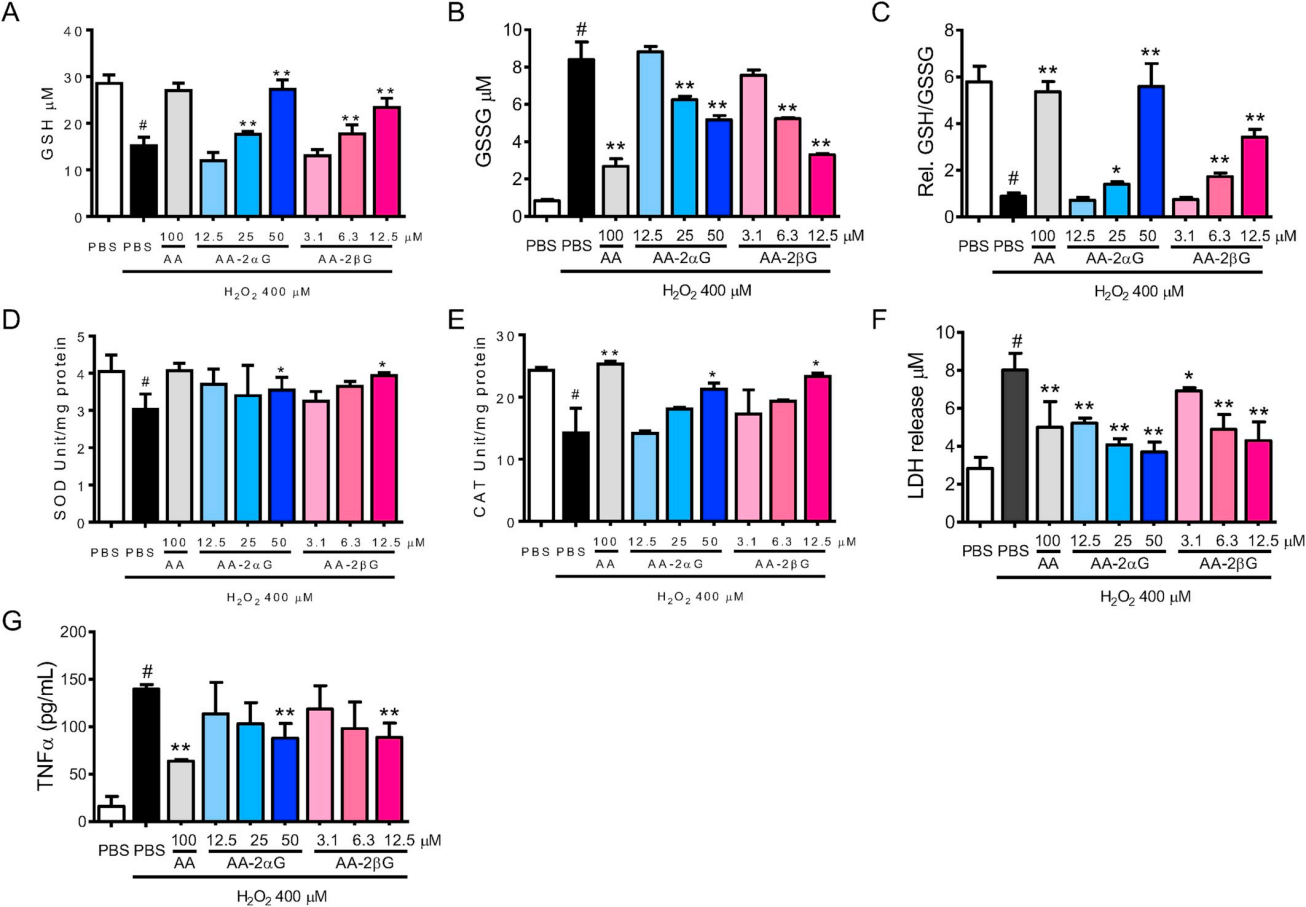
AA, AA-2*α*G, and AA-2*β*G increased the intracellular antioxidant enzyme activity. RAW264.7 cells were pretreated with different of AA, AA-2*α*G, and AA-2*β*G concentrations for 4 h, followed by H_2_O_2_ treatment for another 6 h. The effects on (A) GSH level, (B) GSSG level, (C) GSH/GSSG ratio, (D) SOD level, (E) CAT level, (F) LDH level and (G) TNF-α level released into the culture medium were determined using commercially available kits following the manufacturer's instruction. The results are presented as mean ± SD of the three separate experiments (*, *p* < 0.05 and **, *p* < 0.01 compared with H_2_O_2_ treatment; #, *p* < 0.05 compared with PBS treatment).

Intracellular GSH depletes toxic radicals in acute oxidative stress. The recovery of the intracellular GSH content could relieve oxidative damage. Hydrogen peroxide challenge significantly depleted intracellular GSH level and increased the GSSG level, which decreased the ratio of GSH to GSSG compared with PBS-treated control group (Fig. 2A–C). Treatment with AA, AA-2*α*G, and AA-2*β*G significantly increased the GSH level and decreased the GSSG level, thereby enhancing the GSH-to-GSSG ratio (Fig. 2A–C). AA-2*β*G at 12.5 μM exhibited similar activity in restoring the GSH level but showed low potency in GSSG depletion, and hence increased the ratio of GSH to GSSG as 50 μM AA-2*α*G and 100 μM AA did (Fig. 2A–C). Superoxide dismutase (SOD) is an important metalloenzyme that specifically remove O_2_^−^ anions [26]. Catalase (CAT) catalyzes the decomposition of H_2_O_2_, thereby preventing oxidation [27]. Treatment with AA, AA-2*α*G, and AA-2*β*G ameliorated the suppressive effect of H_2_O_2_ on SOD and CAT activities to levels comparative to those of PBS-treated control (Fig. 2D and E).

AA at 100 μM remarkably suppressed LDH (Fig. 2F) and TNF-α (Fig. 2G) release in H_2_O_2_-treated RAW264.7 cells. AA-2*α*G and AA-2*β*G potently inhibited H_2_O_2_-triggered LDH (Fig. 2F) and TNF-α (Fig. 2G) release at 50 and 12.5 μM, respectively.

### 3.3 AA, AA-2*α*G, and AA-2*β*G prevented CPT-11-induced oxidative stress

**Fig. 3:**
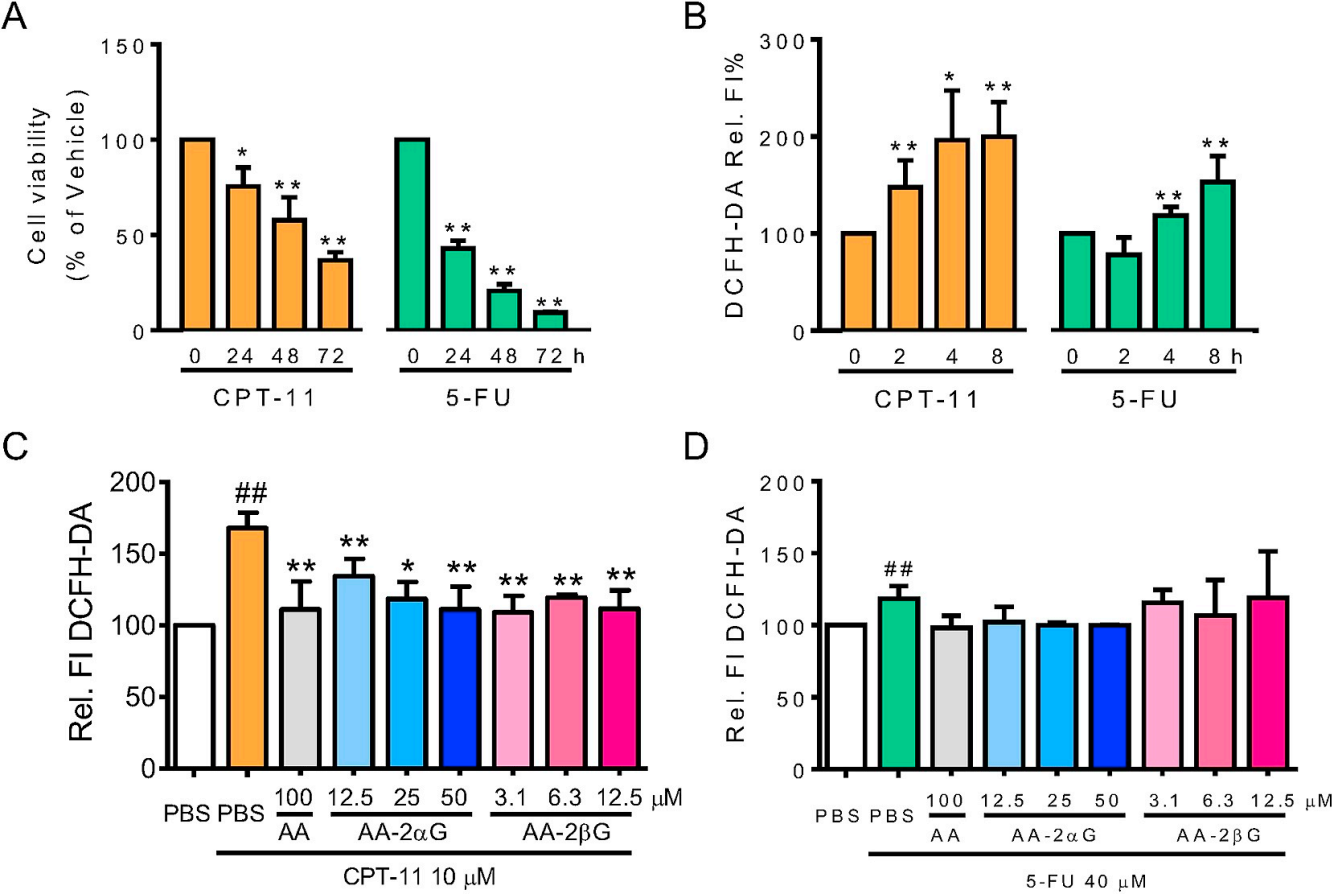
Treatment with AA, AA-2*α*G, and AA-2*β*G prevented CPT-11-induced ROS. (A) Cell viability of RAW264.7 cells treated with 10 μM CPT-11 or 40 μM 5-FU for up to 72 h. (B) ROS generation with CPT-11 or 5-FU treatment was analyzed with DCFH-DA staining. RAW264.7 cells were pretreated with different concentrations of AA, AA-2*α*G, and AA-2*β*G for 1 h, followed by treatment with CPT-11 or 5-FU for the time indicated on the figure. ROS generation in (C) CPT-11 pretreatment for 2 h and (D) 5-FU pretreatment for 4 h were analyzed by DCFH-DA staining. The results are presented as mean ± SD of the three separate experiments (*, *p* < 0.05 and **, *p* < 0.01 compared with H_2_O_2_ treatment; #, *p* < 0.05 compared with PBS treatment).

Then, we extended to determine the antioxidant activities of AA, AA-2*α*G, and AA-2*β*G in chemotherapeutic agents-induced initial oxidative stress. The cytotoxic alkaloid agent CPT-11 at 10 μM and the nucleoside metabolic inhibitor 5-FU at 40 μM inhibited viability of RAW264.7 cells in a time-dependent manner (Fig. 3A–B). CPT-11 triggered remarkable ROS production (Fig. 3B); while 5-FU only induced significant substantial oxidation of DCFH-DA (Fig. 3B). Treatments with AA, AA-2*α*G, and AA-2*β*G significantly decreased CPT-11- but not 5-FU-triggered oxidation of DCFH-DA (Fig. 3C–D).

### 3.4 AA-2*β*G exhibited enhanced the Nrf2 DNA binding activity than AA and AA-2*α*G

**Fig. 4:**
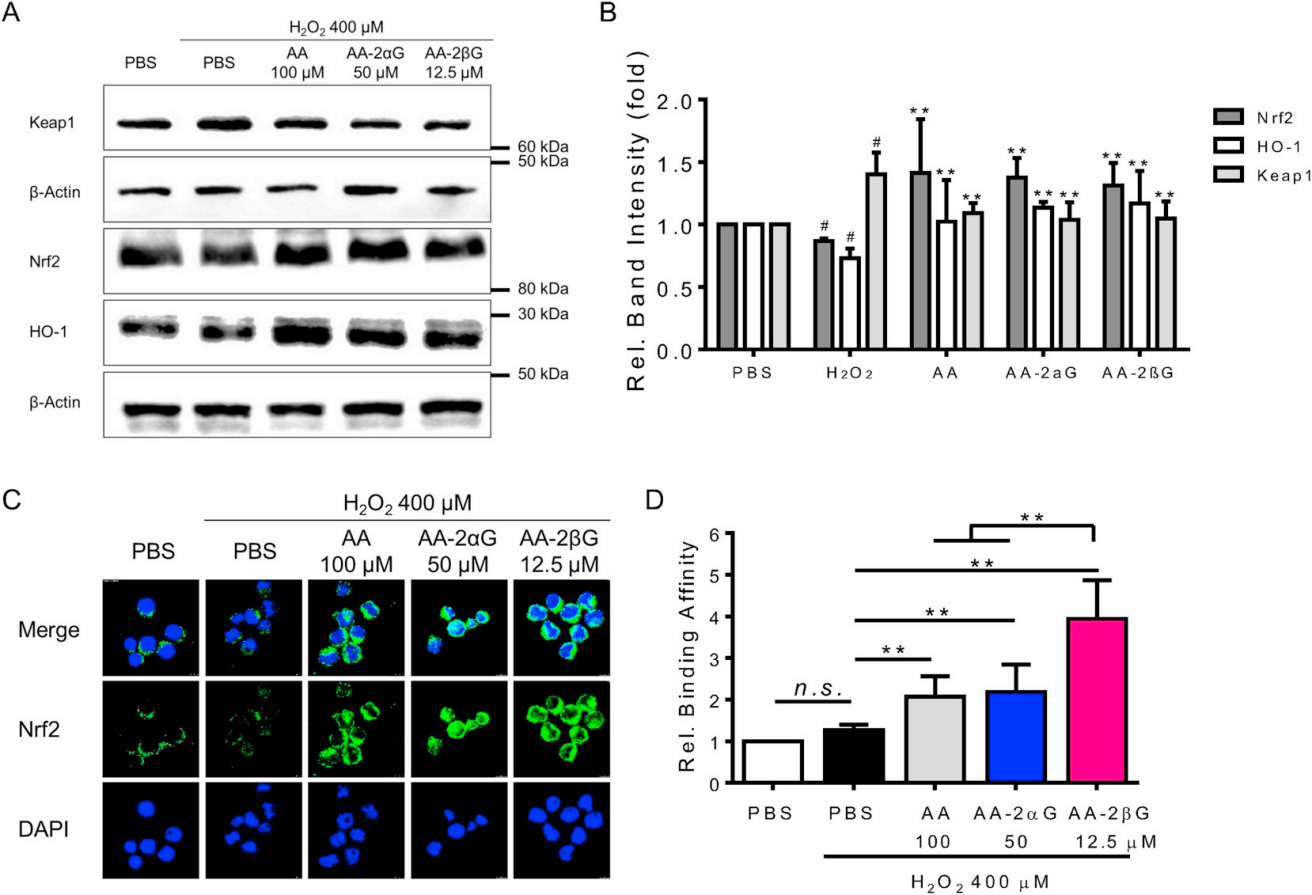
AA, AA-2*α*G, and AA-2*β*G activated the Nrf2 signaling pathway. RAW264.7 cells were pretreated with AA, AA-2*α*G, and AA-2*β*G for 4 h, followed by H_2_O_2_ treatment for another 6 h. (A) The expression levels of Keap-1, Nrf2, and HO-1 in RAW264.7 cells, and (B) the normalized band intensities of Keap-1, Nrf2, and HO-1 compared with that of β-actin. (C) Nuclear translocation of Nrf2 was detected with immunofluorescent staining. (D) Nrf2-DNA binding affinity of the nuclear extract from RAW264.7 cells pretreated with H_2_O_2_ and then incubated with either PBS, AA, AA-2*α*G, or AA-2*β*G. The binding of Nrf2 to its response element was determined using Trans^AM^ Nrf2 kit following the manufacture's recommendation. Results in (A) and (C) represent those of the three separate experiments. Results in (B) and (D) were presented as mean ± SD from three separate experiments (**, *p* < 0.01 compared with H_2_O_2_ treatment; #, *p* < 0.05 compared with PBS treatment; *n. s.*, not significant).

We next determined the effects of AA, AA-2*α*G, and AA-2*β*G on the Keap-1/Nrf2 signaling pathway, which governs the transcription of several downstream antioxidant and detoxifying enzymes to eliminate intracellular radical [28]. The expression Keap-1 level stimulated by H_2_O_2_ was downregulated with AA, AA-2*α*G, and AA-2*β*G treatments (Fig. 4A–B). Meanwhile, AA, AA-2*α*G, and AA-2*β*G significantly reversed the H_2_O_2_-suppressed Nrf2 and HO-1 expression levels (Fig. 4A–B). AA, AA-2*α*G, and AA-2*β*G induced the nuclear translocation of Nrf2 after H_2_O_2_ stimulation (Fig. 4C) and enhanced the binding between Nrf2 and its targeting DNA sequence (Fig. 4D). In particular, AA-2*β*G at 12.5 μM exhibited approximately twofold higher potency in promoting Nrf2-DNA-binding activity than those of 100 μM AA and 50 μM AA-2*α*G (Fig. 4D).

### 3.5 Inhibition of SVCT and GLUT partially abrogated the antioxidant activity and cellular uptake of AA, AA-2*α*G, and AA-2*β*G

**Fig. 5:**
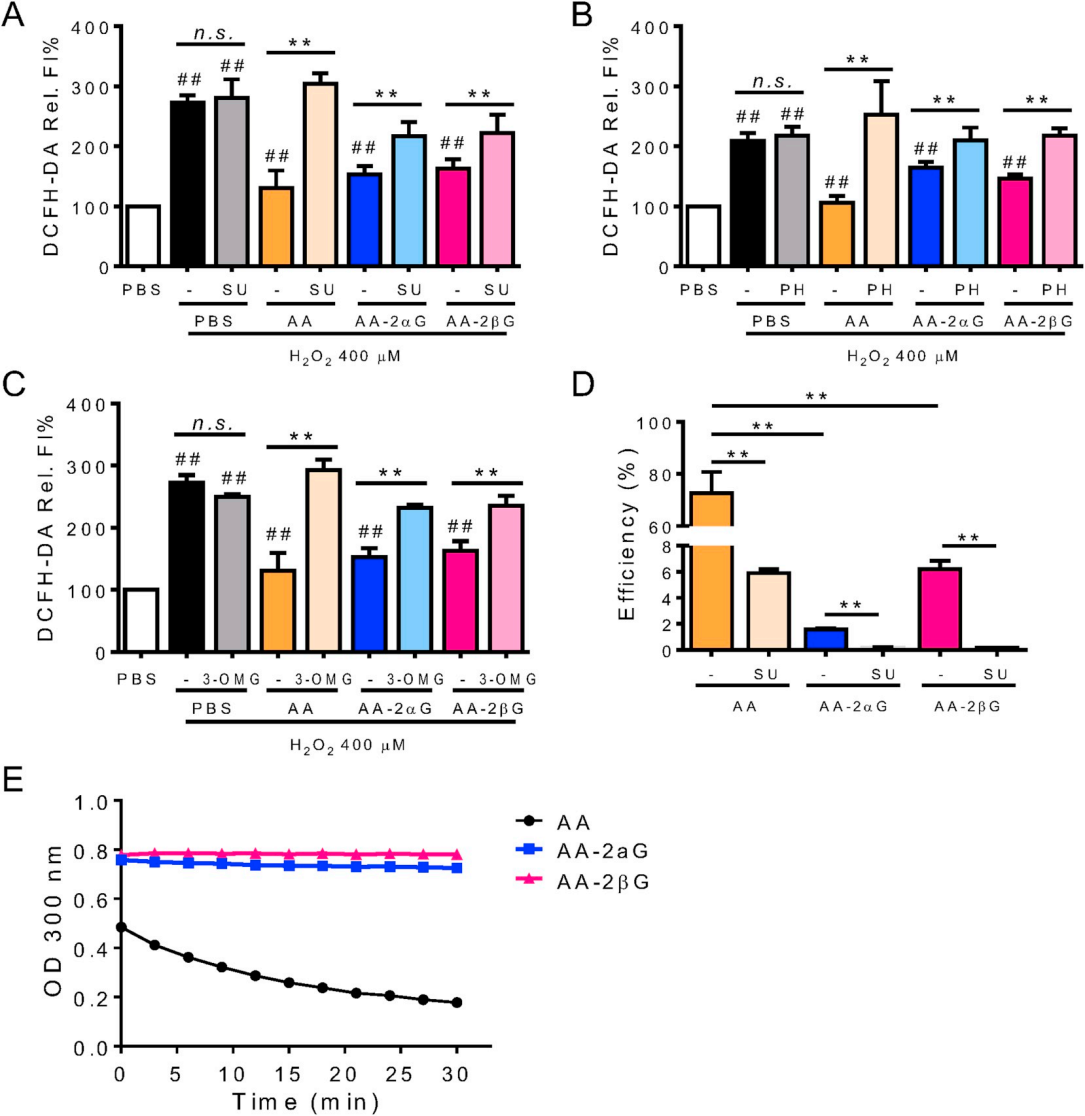
Inhibition of SVCT abrogated the antioxidant activities of AA, AA-2*α*G, and AA-2*β*G. RAW264.7 cells were incubated with SVCT inhibitors (A) 5 μM SU, (B) 10 μM PH, and (C) 10 μM 3-OMG for 20 min and then pretreated with 100 μM AA, 50 μM AA-2*α*G, and 12.5 μM AA-2*β*G for 1 h, respectively. ROS generation was stimulated with 400 μM H_2_O_2_ for 30 min, and determined using DCFH-DA staining. (D) RAW264.7 cells were preincubated with 5 μM SU for 20 min and then treated with AA and its derivative for 1 h similar to (A). The intracellular content of AA, AA-2*α*G, and AA-2*β*G contents were detected by ESI-QTOF-MS/MS. The uptake efficiency was calculated based on the total amount of each compound identified in the cell extract to the amount added in the culture medium. (E) *In vitro* stability of AA, AA-2*α*G, and AA-2*β*G in Fenton reagents for up to 30 min. Results are presented as mean ± SD from three separate times (*, *p* < 0.05 and **, *p* < 0.01 compared with H_2_O_2_ treatment; #, *p* < 0.05 compared with PBS treatment; *n. s.*, not significant).

**Fig. 6:**
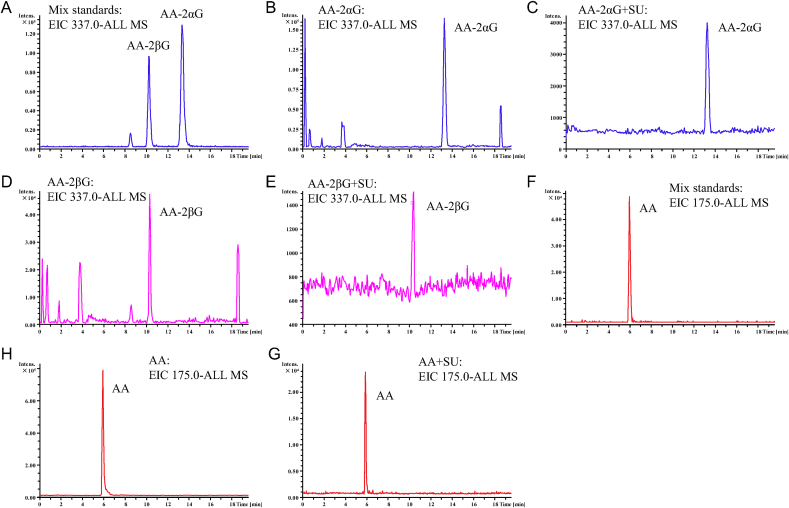
ESI-QTOF-MS analysis spectrum of AA, AA-2*α*G, AA-2*β*G. (A) AA-2*α*G and AA-2*β*G standard. (B) AA-2*α*G in RAW264.7 cell lysate. (C) AA-2*α*G in cell lysate when RAW264.7 cells were pre-treated with 10 μM sulfinpyrazone (SU). (D) AA-2*β*G in RAW264.7 cell lysate. (E) AA-2*β*G in cell lysate when RAW264.7 cells were pre-treated with 10 μM SU. (F) AA standard. (G) AA in RAW264.7 cell lysate. Results are representative of three individual experiments.

Pretreatment with the nonselective SVCT inhibitor SU and GLUT inhibitors PH and 3-OMG partially abrogated the abilities of AA, AA-2*α*G, and AA-2*β*G to decrease the level of oxidized DCFH-DA (Fig. 5A–C). Then, we measured the cellular uptake and possible oxidized product(s) of AA and its derivatives by using ESI-QTOF-MS/MS in RAW264.7 cells. The ESI-QTOF-MS/MS analysis results revealed that 70% of AA added into the culture medium was present in the cell lysate (Fig. 5D), while the uptake efficiency of AA-2*α*G and AA-2*β*G was ≤10% (Fig. 5D). We did not find any oxidized products of AA, or other metabolites of AA-2*α*G or AA-2*β*G in RAW 264.7 cells using ESI-QTOF-MS analysis (Fig. 6). The ferrous iron content suggested that approximately 50% of AA was oxidized after 30 min; while AA-2*α*G and AA-2*β*G remained in their reduced state *in vitro* (Fig. 5E).

## 4 Discussion

In the present study, we reported the excellent antioxidant activity of AA-2*β*G, which is a unique natural AA derivative identified in *L. barbarum*. The antioxidant activity of AA-2*β*G is related to activation of the Keap1/Nrf2 signaling pathway and SVCT-dependent cellular uptake. The presence of the d-gluocosyl moiety on AA-2*α*G and AA-2*β*G prolonged their free radical scavenging activity but reduced their cellular uptake compared with AA. The *β*-configuration of AA-2*β*G also promoted the Nrf2-DNA-binding activity.

Antioxidants are used as food supplement to protect against the development of many types of aging-related diseases, maladaptive inflammation-induced macrophage death [29], and hematopoietic toxicity of chemotherapeutic agents. AA is one of the most easily accessible antioxidants from natural resources. AA exerts its scavenging activity mainly through direct redox reaction to restore the intracellular GSH pool [5]. The addition of the glucosyl group to AA-2*α*G and AA-2*β*G replaced the potentially oxidizable hydroxyl group on the C-2 position of AA. The lack of C-2 hydroxyl group may decrease the potent scavenging activities of AA-2*α*G and AA-2*β*G on GSSG, SOD, and CAT levels in comparison to AA (Fig. 2A–E) [20]. This is in line with previous finding that baicalin exhibited higher radical scavenging activity than its aglycone baicalein [30]. AA-2*α*G and AA-2*β*G were not metabolized to AA (Fig. 6). This may be due to the reason that RAW264.7 cells bare relatively low expression levels of α- or β-glucosidase (data not show), thus could breakdown the glycosidic bond. These results suggested that AA-2*α*G and AA-2*β*G may not quench free radicals through AA as intermediate but either through the activation of the cellular defense system or formation of covalent adduct with free radicals.

High AA concentration (10 g/day) is required to achieve the antioxidant capacity in overcoming its fast oxidation rate in the clinic [31]. High AA concentration is associated with increased kidney stone incidence and considerable renal, cardiac, and metabolic toxicity [8]. Several AA analogs have been synthesized to reduce the oxidation rate and increase stability. 6-O-Palmitoylascorbate, a lipophilic AA derivative, inhibits DNA damage and apoptotic cell death-induced by X-ray in submillimolar concentration [32,33]. The amphipathic AA derivative, 3-*O*-laurylglyceryl ascorbate, maintains the free radical scavenging activity through the peroxisome proliferator activated receptor-γ and Nrf2 signaling pathways [34]. AA-2*α*G, which is widely used in the cosmetic and food industry as an antioxidant supplement, exhibits similar activity to that of AA in preventing H_2_O_2_-induced oxidative stress in dermal fibroblasts [35,36]. These AA derivatives are more stable than AA, but their potency are also similar to that of AA. The concentration required for AA-2*β*G to reduce direct DCFH-DA oxidization triggered by H_2_O_2_ or CPT-11 was approximately 25% of that required for AA-2*α*G (12.5 μM vs 50 μM) or 12% (12.5 μM vs 100 μM) required for AA (Fig. 1, Fig. 3C). But AA and its derivatives exhibit no effect on the initial substantial ROS induced by 5-FU (Fig. 3D), which was mediated through inhibition of DNA and RNA synthesis [37]. AA, AA-2*β*G, or AA-2αG did not affect cell viability alone as detected by MTT assay (Fig. 1B) but abrogated H_2_O_2_-induced LDH and TNF-α release (Fig. 2F–G). MTT and LDH release assays are primarily based on intracellular redox reactions. Thus, the results may be affected by the antioxidant properties of AA, AA-2βG, or AA-2αG. But fortunately TNF-α secretion was indirectly affected by intracellular ROS level. Therefore, AA-2*β*G or AA-2αG exhibits enhanced free radical-scavenging activity per molecule against cell death compared with AA (Fig. 2F–G).

Antioxidants directly scavenge free radicals or reduce oxidative stress through restoration of the antioxidant defense mechanism. The fast oxidation rate suggested that AA directly interacts with free radicals (Fig. 5E). DHA, which is the oxidized product of AA, induces cell death in colorectal cancer carrying KRAS and BRAF mutation by deactivating glyceraldehyde 3-phosphate dehydrogenase [5]. The cytotoxic activity of DHA is also dependent upon its ability to induce oxidative stress by oxidizing GSH to GSSG [5,20]. Our results demonstrated that the controversial effect of AA as antioxidant in the clinic may be due to the metabolism and genetic background of the disease it was used to treat, and the high dose required. AA-2*α*G activates the SIRT1 signaling pathway to delay the pathogenesis of dermal fibroblasts from H_2_O_2_-induced oxidative damage and cellular senescence [36]. AA-2*α*G maintains the integrity of gastric epithelial cells from *Helicobacter pylori*-initiated mitochondrial death signaling [38]. We also proposed that the Keap-1/Nrf2 signaling is at least partially activated with AA, AA-2*α*G, and AA-2*β*G treatment (Fig. 4).

SVCT and GLUT are generally recognized as rate limiting factors and universal transporters for AA in different cell types [5,39]. Intracellular AA also increases SVCT expression and its membrane localization, which further promotes AA uptake [39,40]. Inhibition of SVCT or GLUT abrogated the antioxidant activity (Fig. 5A–C) and reduced the cellular uptake (Fig. 5D) of AA, AA-2*α*G, or AA-2*β*G (Fig. 5A–D). This is in line that glucose conjugation did not change the physical or chemical activities of platinum (II) drugs and maintained their cellular target and active pharmacophore [41,42]. The concentration of AA-2*β*G to reduce oxidized DCFHDA was only 25% to that of AA-2*α*G (compare 12.5 μM–50 μM on Fig. 5D); while, the cellular uptake efficiency of AA-2*β*G was approximately threefold to that of AA-2*α*G (Fig. 5D). Thus, the intracellular AA-2*α*G and AA-2*β*G are of similar concentrations. AA-2*β*G exhibited the same direct radical scavenging activity in the Fenton reagent (Fig. 5E), two fold activity in promoting Nrf2-DNA binding (Fig. 4D), and approximately 50% activity in restoration of intracellular GSH/GSSG (Fig. 2C) to AA-2*α*G. Thus, the *β*- but not the *α*-configuration of the glucosyl group favors activation of antioxidant genes instead of direct radical scavenging activity. The stability of AA-2*α*G and AA-2*β*G was much higher than that of AA in the Fenton reagent (Fig. 5E), thus less AA-2*α*G or AA-2*β*G is required to reduce free radicals than AA (Fig. 1, Fig. 3C).

In summary, the study showed the following results: (1) the reactivity against free radical followed the order of AA-2*β*G > AA-2*α*G > AA; (2) AA-2*α*G and AA-2*β*G were not metabolized into AA in RAW264.7 cells, thereby suggesting that their antioxidant activity may not require AA as intermediate; (3) AA-2*α*G and AA-2*β*G shared the same cellular uptake transporters as AA; and (4) the glucosyl moiety and its configuration on AA-2*β*G increased stability and enhanced the Nrf2-DNA-binding affinity. Here, AA-2*β*G can be used as a highly reactive antioxidant for the prevention of chemotherapeutic agents-induced damage and other free radical-triggered diseases.

## Declaration of conflicts

The authors declare no conflict of interests.
